# Pediatric-Onset Multiple Sclerosis (POMS) and Epilepsy: Exploring Etiological Complexity—Outcomes from a Single-Center Experience

**DOI:** 10.3390/children12050631

**Published:** 2025-05-14

**Authors:** Alice Denisa Dică, Dana Craiu, Catrinel Iliescu, Marcel-Alexandru Găină, Carmen Sandu, Cristina Pomeran, Diana Bârcă, Niculina Butoianu, Carmen Burloiu, Ioana Minciu, Alexandra-Maria Găină, Dana Șurlică, Cristina Moțoescu, Oana Tarța-Arsene, Cristina Cazacu, Andreea Badea, Alexandru Ștefan Niculae, Daniela Adriana Ion

**Affiliations:** 1 “Prof. Dr. Alexandru Obregia” Clinical Hospital of Psychiatry, 041914 Bucharest, Romania; alice.dica@drd.umfcd.ro (A.D.D.); diana.barca@umfcd.ro (D.B.); maria-cristina.calusaru@drd.umfcd.ro (C.C.); alexandru-stefan.niculae@rez.umfcd.ro (A.Ș.N.); 2 Department of Pediatric Neurology, Faculty of Medicine, “Carol Davila” University of Medicine and Pharmacy, 020021 Bucharest, Romania; daniela.ion@umfcd.ro; 3 3rd Medical Department–Psychiatry, “Grigore T. Popa” University of Medicine and Pharmacy, 700115 Iași, Romania; alexandra_gaina@email.umfiasi.ro; 4 Department of Pathophysiology, National Institute for Infectious Diseases “Prof. Dr. Matei Balș”, 021105 Bucharest, Romania

**Keywords:** pediatric multiple sclerosis, epilepsy, EEG, seizures, EDSS

## Abstract

This article examines the complex relationship between seizures, epilepsy, and multiple sclerosis (MS) in pediatric patients, based on detailed findings from a single-center study. **Background**: Although multiple sclerosis is primarily recognized as an adult-onset disease, its occurrence in children presents distinctive challenges, especially related to seizure disorders. **Methods**: We reviewed 120 pediatric MS patients evaluated over 7 years; six of these (5%) experienced seizures (including one case of acute status epilepticus), and five were diagnosed with epilepsy according to the latest International League Against Epilepsy (ILAE) classification. This study aimed to evaluate the occurrence rates and types of seizures while investigating their management strategies in this specific group. **Results**: Through a detailed case analysis and patient follow-up, we identified key factors contributing to seizure onset and explored implications for treatment and care. In our cohort, children with MS and seizures showed a higher risk for disease progression and greater cumulative disability, evidenced by a significantly higher last Expanded Disability Status Scale (EDSS) score (after a minimum 2-year follow-up) in the seizure group (*p* < 0.006). The analysis recognized early MS onset and highly active disease types as further risk factors that led to worse health outcomes. **Conclusions**: Genetic causes of epilepsy in children are common and may interact with MS-related inflammation in the same patient; our observations underscore the need to investigate how these two conditions interact. This work contributes to the broader understanding of epilepsy comorbid with MS among pediatric patients, seeking to facilitate the creation of improved interdisciplinary clinical practices in pediatric neurology.

## 1. Introduction

Understanding the intricate link between neurological diseases—especially seizures, epilepsy, and multiple sclerosis (MS) in pediatric populations—has drawn increasing interest recently [1,2,3]. This study sought to investigate these interconnected conditions in a cohort of 120 children with MS, six of whom (5%) had seizures and epilepsy.

Seizures, defined as abrupt, uncontrolled electrical disruptions in the brain, are a frequent manifestation in children with neurological diseases and can greatly reduce quality of life [4,5]. Epilepsy, characterized by recurrent unprovoked seizures, often coexists with other neurological illnesses, including MS, a chronic central nervous system disorder that can cause diverse neurological symptoms [4,5,6,7].

The group of children who develop multiple sclerosis (known as pediatric-onset MS or POMS) forms a distinct subgroup that presents with unique clinical characteristics and different challenges compared to those faced by adults.

Epilepsy is one of the most common chronic neurological disorders in childhood, particularly in the first 10 years of life, affecting around 0.5–1% of children worldwide [8]. The reported prevalence is about 17.3 per 1000 children (range 3.2–44), with an incidence of 41–187 per 100,000 person-years [9] and approximately 2.5 per 1000 in some studies [8]. In contrast, MS is very rare in pediatric ages; less than 5% of all MS cases begin before 18 years old [10,11,12,13].

Although these two conditions seldom appear in the same individual (only about 2–3% of MS patients have comorbid epilepsy), this percentage is higher than in the general population [14,15,16,17] and seems to be even greater in younger patients (reported at 5–10% in pediatric MS cohorts) [18,19].

When examining the mechanisms that might link MS and epilepsy in children, the situation becomes complex and remains poorly understood. It is unclear whether there is a direct association or if they co-occur as independent conditions. Multiple pathophysiological mechanisms have been suggested, which provide incomplete explanations for the development of epilepsy in patients with MS. These include neuroinflammation due to demyelinating lesions in both white and gray matter [1,20,21]; global cerebral atrophy, especially involving the hippocampus and other temporal lobe structures (key epileptogenic areas) [1,21,22,23]; impairments in blood–brain barrier (BBB) function, leading to heightened neuronal excitability [24,25,26]; and the proinflammatory role of excessive glutamate signaling [23,25]. Despite these factors, many MS patients appear to have a degree of “resilience” to developing seizures [2,19,27].

The gray matter regions that make up the hippocampus and insula together with the frontal and temporal lobes play a central role in epilepsy because they are prone to generating seizures. These seizures trigger inflammation because of the activation of abnormal neuronal circuits [1,20,21]. The inflammatory response can result in blood–brain barrier dysfunction and glutamate-mediated excitotoxicity through glutamate, while promoting epileptogenic processes [2,24,26]. Meanwhile, the known etiologies of epilepsy in children are diverse; genetic mutations and structural abnormalities (malformations of cortical development, e.g., focal cortical dysplasia [28] or tumors) are among the most common causes, alongside metabolic, immune [29], infectious, and acute injury causes (head trauma, stroke) [30,31,32,33]. Notably, genetic and autoimmune influences can coexist. As can be seen, inflammation, BBB disruption, and glutamate-mediated excitability are several mechanisms that are common to both MS and epilepsy.

The primary aim of this study was to highlight different possible scenarios that may appear when we have a child with MS and epilepsy regarding the seizures’ etiology, because, sometimes, it is difficult to establish the exact correlation between these two conditions (whether one produces the other). This is due to the fact that, in the case of children with epilepsy, genetics play an important role, and two disorders may coexist, with varying degrees of influence on each other. Secondarily, we found that, in our study cohort, patients with both MS and epilepsy had a significantly higher last EDSS score (indicating greater disability accumulation after at least 2 years of monitoring; *p* < 0.006), compared to those without epilepsy. Recognizing these patterns may help to refine the diagnostic and treatment approaches for this unique patient group.

## 2. Materials and Methods

The present retrospective observational study covered a 7-year period (January 2018–December 2024). We reviewed the medical records of pediatric patients (<18 years) with MS who were diagnosed, monitored, and treated in the Pediatric Neurology Department of “Prof. Dr. Alexandru Obregia” Clinical Hospital of Psychiatry in Bucharest, Romania, the largest tertiary pediatric neurology center in the country.

A total of 120 subjects were selected, who met the 2017 revised McDonald criteria (2017) for MS diagnosis. Among these, 6 children (3 boys and 3 girls; median age 9.8 years, range 4.6–15.3 years) experienced seizures or epilepsy. Detailed data on their epileptic events (age at seizure onset; timing relative to MS onset; seizure type, frequency, and duration; whether an epilepsy diagnosis was established; anti-epileptic treatment; and whether seizure control was achieved) are summarized in Table 1. Seizure and epilepsy types were classified according to the latest ILAE recommendations (the 2017 ILAE seizure classification and the 2022 ILAE epilepsy classification).

**Figure A1:**
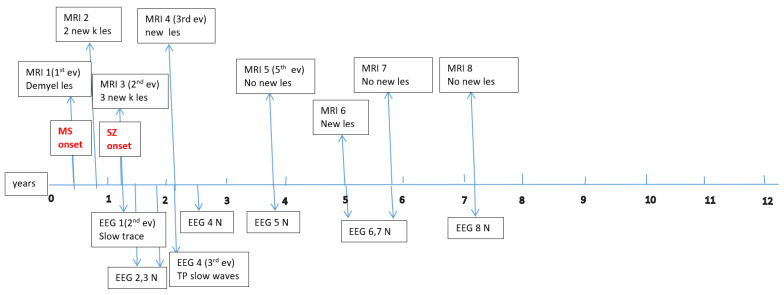
P1 Brain MRI and EEG frequency and aspects.

**Figure A2:**
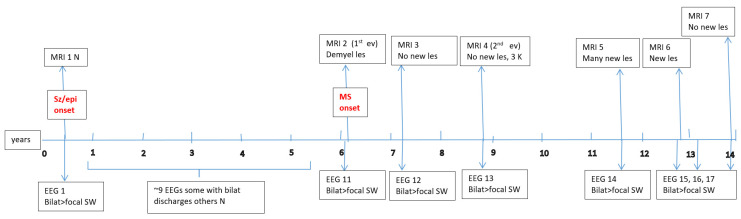
P2 Brain MRI and EEG frequency and aspects.

**Figure A3:**
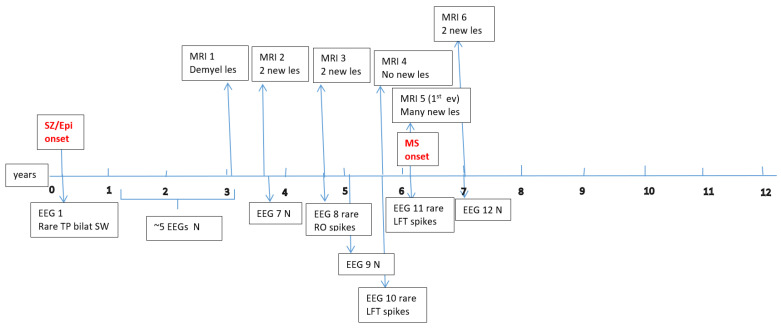
P3 brain MRI and EEG frequency and aspects.

**Figure A4:**
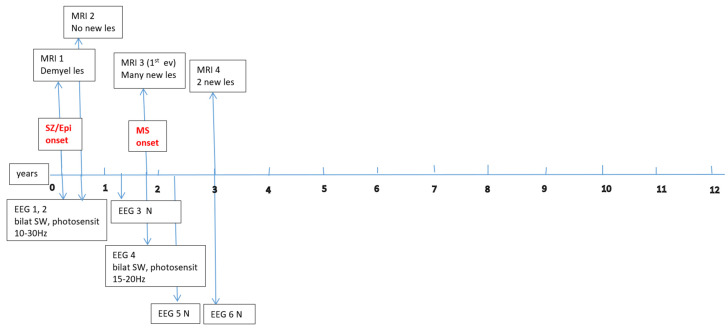
P4 brain MRI and EEG frequency and aspects.

**Figure A5:**
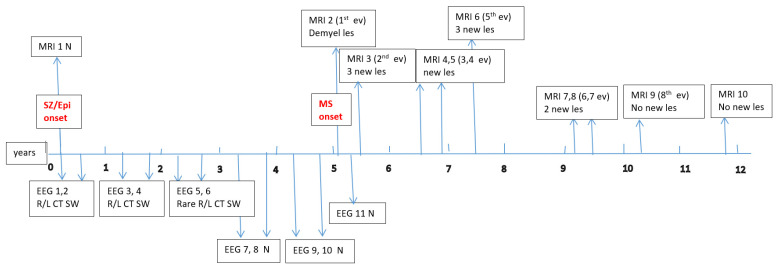
P5 brain MRI and EEG frequency and aspects.

**Figure A6:**
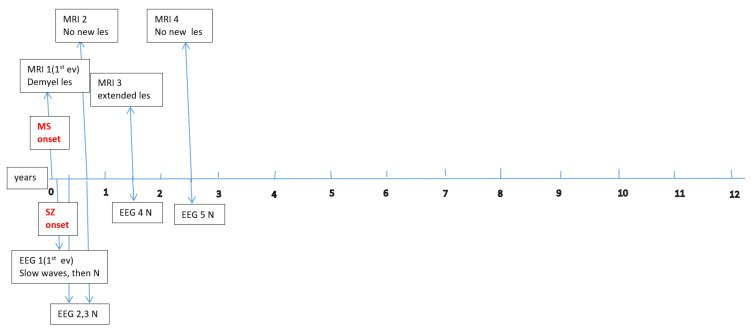
P6 brain MRI and EEG frequency and aspects.

Data about each patient’s electroencephalography (EEG) and brain magnetic resonance imaging (MRI) findings at MS onset and during disease evolution were collected. Figure A1, Figure A2, Figure A3, Figure A4, Figure A5 and Figure A6 (see Appendix A) show these EEG and MRI data for the six patients. For EEG recordings, the international 10–20 system was used with a double banana montage and additional ECG channel; all 6 patients had at least one prolonged EEG (3–4 h) that included both awake and sleep recording. Brain MRI scans were performed on a 1.5T scanner, without and with gadolinium contrast.

The statistical analysis included the Mann–Whitney U test to assess differences between groups due to deviations from a normal distribution, as confirmed by Shapiro–Wilk testing. The variables analyzed were clinical metrics such as age at MS onset and diagnosis; the durations between the onset, diagnosis, and initiation of DMT; and the durations of MS monitoring and DMT treatment throughout the study, as well as annualized relapse rates (ARR) before and after treatment at intervals of 1, 2, and 3 years. No statistically significant differences emerged for these measures, except for the final EDSS score, which was significantly worse in patients with epilepsy compared to those without epilepsy. Comprehensive statistical outcomes are detailed in Appendix B.

Although there was a large discrepancy in the group sizes (MS with seizures n = 6; MS without seizures n = 114), we compared disease progression between these groups using annual EDSS scores and observed that there was a statistically significant correlation with the last EDSS score (Table 2 and Table 3).

For the statistical analysis and data processing, we used JASP 0.19. Categorical comparisons were performed using the chi-squared test with Yates’ continuity correction. A *p*-value < 0.05 was considered statistically significant.

Ethical approval for this study was obtained from the hospital’s ethics committee, and written informed consent was obtained for all patients (from parents or legal guardians for minors).

## 3. Results

Our main focus was to describe the particular characteristics of a small group of patients with MS and epilepsy.

### 3.1. Concerning Seizures and Epilepsy

Among the six children, seizure onset occurred before 10 years of age in three patients (50%); one child experienced their first seizure at age 10 (16.66%); and the remaining two had seizure onset during adolescence (33.3%). In four patients (66.66%), seizures began before the onset of MS. These four were diagnosed with epilepsy years before MS was recognized, with a median interval of 4.7 years (range 1.8–6.5 years) between epilepsy onset and MS onset. By contrast, in the two patients whose first seizures occurred after MS onset, the latency was much shorter and seemed temporally associated with MS relapses—one had a seizure 2 weeks after the first MS episode, and the other had seizures ~9 months after MS onset as part of the second relapse. In the latter patient, it was also the only seizure, and it took the form of a focal prolonged seizure with impairment in consciousness at the end of the event, followed by sloth, left central facial palsy, and a transient bipyramidal syndrome lasting a few days. Although this was considered an acute episode, given the prolonged seizure and high risk of recurrence, the child received anti-seizure medication for 6 months. This intervention resulted in a good outcome, with no further seizures and a normal EEG during follow-up.

The other teenager who had seizures shortly after his first MS relapse was treated with anti-epileptic drugs (AEDs) for two years, because his repeated focal alternate seizures eventually led to a diagnosis of focal structural epilepsy (with a probable autoimmune etiology). Furthermore, in this case, the evolution was favorable, and he has now been seizure-free for about 3 years and has discontinued AEDs.

Among the subjects whose seizures predated MS, two appeared to meet the criteria for a genetic form of epilepsy-self limited focal epilepsy with centro-temporal spikes (SeLECTS) and idiopathic generalized epilepsy (IGE), respectively, according to the recent ILAE seizures and epilepsy classification, although any immune contribution in these cases remains unclear. The other two patients in this subgroup presented a more complex situation: they continued to have uncontrolled, pharmacoresistant seizures despite polytherapy (three to four anti-seizure drugs), with multiple seizure types and persistent EEG epileptiform discharges. Notably, one had a history of simple febrile seizures in early childhood (before epilepsy onset), and another had a family history of epilepsy; both of these patients experienced an increase in seizure frequency after MS onset, suggesting a possible combination of genetic and autoimmune etiologies. In two of the four patients whose seizures started before MS, the early brain MRI scans (at the time of initial seizures) were normal.

Four children (67%) had a history of at least one simple febrile seizure in infancy; one patient had a first-degree relative with adolescent-onset epilepsy and another had a first-degree relative with adult-onset MS. At the last follow-up, four of the six children (67%) achieved good seizure control, with three of them discontinuing any anti-seizure medication, whereas two patients (33%) had ongoing drug-resistant epilepsy. Table 1 provides these details for each patient. Each child underwent multiple EEG studies, especially during periods of disease worsening or medication changes. Approximately one-third of all EEGs were prolonged recordings (including sleep), while the others were standard awake EEGs with activation procedures (hyperventilation and intermittent photic stimulation). Figure A1, Figure A2, Figure A3, Figure A4, Figure A5 and Figure A6 (see Appendix A) provide illustrative EEG and MRI findings for each patient.

All five patients with epilepsy exhibited epileptiform discharges on EEG at the onset of their epilepsy; subsequently, only the two patients with persistent seizures continued to demonstrate EEG abnormalities over time. The patient presenting with status epilepticus initially showed slow EEG background activity, which normalized in later EEG recordings.

Due to the group heterogeneity regarding the potential seizure etiologies and MRI findings across all six patients, characterized by multiple extensive, confluent lesions, it was difficult to establish a clear pattern for the localization of epileptogenic areas. Even in the two children where an autoimmune etiology was presumed responsible for provoking the seizures, the precise localization of the epileptogenic focus remained elusive, considering inflammation and demyelination per se as the seizures’ sources.

### 3.2. Concerning MS

Concerning the MS presentations in these six patients, the initial clinical features of MS were as follows: hemiparesis in four cases (66.7%); a combination of hemiparesis, cerebellar syndrome, and multiple cranial nerve involvement in one case; and a sensitivity disturbance in one case. The age at MS onset was <10 years in one patient, 10–12 years in two patients, and >12 years in three patients. Two patients had an initial presentation consistent with an isolated clinical syndrome (ICS), while four patients had a relapsing–remitting MS course from onset. Oligoclonal bands in cerebrospinal fluid were positive in four patients (one described as “intensely positive”) and negative in two. The two ICS patients has experienced only a single MS attack to date, whereas the other four have had multiple relapses (two, four, seven, and eight acute events, respectively, over their disease courses). All six patients received disease-modifying therapies (DMTs) for MS: four patients were treated with a single DMT (two on moderate-efficacy injectables like interferon-beta and two on high-efficacy therapies like fingolimod), and the remaining two patients required escalation through multiple DMTs (they started with intravenous immunoglobulin during early childhood, when DMTs were contraindicated due to their age, and then switched to interferon and later to fingolimod, which are the therapies available in our country for pediatric MS). Figure A1, Figure A2, Figure A3, Figure A4, Figure A5 and Figure A6 (see Appendix A) illustrate the burden and characteristics of the demyelinated brain lesions in these patients, which varied according to the disease activity (relapse count), particularly prior to initiating DMTs.

All six patients have been followed up with for at least two years from MS diagnosis. We sought to determine whether the occurrence of epilepsy could be a risk factor for more rapid MS progression and disability accumulation. In our cohort, the EDSS began to diverge from 0 (no disability) after 2–3 years of disease in those with epilepsy. A comparative analysis of annual EDSS progression between MS patients with epilepsy and those without showed that, by the last follow-up, the epilepsy group had significantly higher EDSS scores on average. As noted, the difference in outcome was statistically significant (*p* < 0.006 for the latest EDSS value comparison).

## 4. Discussion

These findings fulfil the main goal of this study: to demonstrate that juvenile pediatric patients with MS and epilepsy constitute a heterogeneous and complex group, making it challenging to define the precise relationship between these two disorders. The inflammatory process in MS is known to be more pronounced in children [34,35] than in adults, and an earlier onset of MS implies a longer duration of disease and a greater cumulative burden of cognitive and motor deficits [34,35].

Epilepsy remains one of the most prevalent pediatric neurological disorders (“a childhood disease”) [6,30,31,32,33]. Genetic and structural etiologies are the most common causes of epilepsy in the pediatric population; therefore, when epilepsy arises in a child who also develops MS, we cannot rule out a possible link with genetic–autoimmune origins, common to their age group. Zuo et al. used a two-sample Mendelian randomization analysis to provide evidence of a bidirectional causal relationship between multiple sclerosis and epilepsy. Specifically, genetic susceptibility to MS increases the risk for epilepsy (particularly generalized epilepsy), and, conversely, epilepsy significantly elevates the risk of developing MS [36].

In our series, we indeed suspect that, in some patients, the epilepsy was primarily genetic (with inflammation from MS often contributing as an aggravating factor), whereas, in others, the epilepsy seemed to be directly related to MS (immune-mediated).

In the young, developing nervous system, the intersection of MS and epilepsy in the same individual raises critical questions about how these conditions interact and to what extent they influence one another’s courses. Demyelinating lesions that are active and localized juxtacortically, as well as cortical lesions, have the potential to disrupt neural circuits and may trigger seizures [5]. MS lesions give rise to an inflammatory response through cytokine release and, together with the disruption of the blood–brain barrier that occurs during relapses [26], can lower the seizure threshold. Further studies are needed to confirm this hypothesis and to better understand the mechanisms involved.

In our study, 33% of the patients experienced their initial seizure within 2 weeks to 9 months from MS onset, which indicates that seizures due to inflammatory processes tend to cluster temporally early during disease progression. Multiple studies show that seizures in MS patients typically occur early in the disease course, when the inflammatory activity reaches its peak [37]. Some studies show seizures appearing before any demyelinating symptoms, which suggests that seizures might serve as the first sign of CNS inflammation in certain patients [38]. The clinical implications of these findings are significant, suggesting that clinicians should pay attention to early seizures in MS patients, which may serve as early markers of acute inflammatory activity within the central nervous system. This could prompt early neuroimaging and fast anti-inflammatory interventions to prevent subsequent neurological deterioration, thus improving long-term outcomes.

This calls for a multidisciplinary perspective—pediatric neurologists specialized in autoimmune disorders, epileptologists, immunologists, and geneticists need to collaborate to untangle the contributions of neuroinflammation, genetics, and other factors. Future multi-center research on larger pediatric cohorts is needed to clarify these interactions.

Answering the above-raised questions could guide clinicians in choosing appropriate treatments for MS and seizures in such patients. Furthermore, some anti-epileptic drugs may not be ideal in the context of MS-related seizures (for example, certain AEDs might aggravate seizures [39,40], while some DMTs for MS appear to also help in managing seizures [34,35]). For example, the DMT glatiramer acetate significantly reduced the seizure frequency and exhibited protection against demyelination in a rat epilepsy model, suggesting therapeutic potential through interactions with MS-related pathologies and anti-epileptic mechanisms involving myelin sheath protection [39]. Another DMT, fingolimod, demonstrated potential in reducing inflammation and demyelination, enhancing remyelination through increased oligodendrocyte progenitor recruitment and differentiation; thus, it may influence the seizure susceptibility and interaction dynamics with anti-epileptic drugs via its direct neurological effects and modulation of CNS inflammation [40]. On the other hand, a meta-analysis published in 2024 by Pozzilli et al. [2] states that the use of sphingosine-1-phosphate receptor (S1PR) modulators (including fingolimod) as DMTs increases the seizure risk significantly more than a placebo or other treatment options for MS patients. Patients treated with S1PR modulators exhibited a seizure incidence rate that was 2.45 times higher than that of the control group. This suggests that neurologists must exercise increased awareness and careful supervision when starting S1PR modulators in pediatric MS patients and that monitorization for seizures and treatment plans need to be adapted as required.

Regarding our study, patients 1 and 5 did not receive AEDs and DMTs simultaneously; they initially received AEDs, with the introduction of DMTs occurring at least three years later. We do not consider the two intravenous immunoglobulin treatments received by patient 1 as DMTs. The three patients treated with levetiracetam (patients 1, 4, and 6) demonstrated good seizure control without MS progression. Additionally, seizures did not recur upon introducing DMTs, despite two of the patients receiving fingolimod. Regarding patients 2 and 3, in which we suspected an underlying genetic–autoimmune etiology, they exhibited treatment-resistant epilepsy of presumed genetic origin, with persistent seizures despite multiple AED regimens. MS subsequently emerged, accompanied by persistent or worsening seizure activity. Although no further MS relapses occurred following the initiation of DMTs, both patients exhibited an aggressive MS phenotype characterized by multiple lesions. Consequently, we anticipate that more potent DMTs (high-efficacy therapies—HETs) may soon be necessary for the better management of the autoimmune activity and potentially seizures. We hypothesize that the coexistence of these two severe conditions in these patients leads to potential mutual exacerbation, increasing the clinical severity of each disorder.

In our study, two patients seemed to have seizures that were directly related to their MS disease activity, whereas, in the two other patients, the seizures were likely due to underlying primary genetic epilepsy, with MS as an additional factor. We hypothesize that, even in those presumed genetic cases, the superimposed neuroinflammation of MS might influence the epilepsy course (for example, making seizures more frequent or difficult to control). Conversely, for the last two patients, we suspect the true convergence of etiologies—an interaction between genetic predisposition and autoimmune demyelination, contributing jointly to their epilepsy—which could explain their particularly challenging courses. However, determining the exact contribution of each factor will require further research, potentially including genetic testing and immunological profiling, which were limitations in our study.

Regarding disease progression, our finding that epilepsy may be a risk factor for increasing disability in pediatric MS aligns with some reports in adult MS populations that have noted worse outcomes in MS patients with seizures [2,3,17]. Grothe et al. conducted an analysis of German MS patients that revealed that MS patients with epilepsy showed higher EDSS scores at initial disease onset and experienced more rapid disability progression and greater cumulative disability than MS patients without epilepsy [16]. Similarly, Saridas et al. emphasized that epilepsy in MS correlates with higher disability levels (mean EDSS 4.07 vs. 2.1), a greater frequency of lesions in the temporal and thalamic regions, and increased brain atrophy, suggesting neurodegeneration as a key underlying factor in epilepsy-related disability progression and reinforcing the link between epilepsy and enhanced disability in MS [41].

On the other hand, other studies on pediatric MS found that comorbid epilepsy did not significantly affect MS outcomes [19]. Research findings show that some disagreement exists. According to a recent review conducted by Kavčič and Hofmann in 2024 [19], children who experience seizures generally show similar long-term MS outcomes compared to those who do not experience seizures.

We underline that, in our study, epilepsy appeared to be a risk factor for a higher last EDSS score and disability accumulation, and we evaluated it in relation to other risk factors that influenced the outcome: a young age at MS onset and a long disease course, as well as the fact that most of these patients had a very active form with many relapses, especially before DMT onset. Therefore, while epilepsy might independently worsen the MS prognosis, it often occurs in the context of aggressive MS. Due to the small sample size and single-center applied study design, which constrain the generalizability of our results, we state that the findings of this research are preliminary and require validation through extensive multicenter studies targeting wider pediatric MS populations to confirm whether seizures themselves drive faster progression or whether they are simply a marker of a more severe disease phenotype.

A major limitation of our study was the lack of genetic and specific immunological profiling data within our cohort, mainly due to the financial burden, as parents support the costs of specific genetic tests in Romania.

While it may not substantially impact the therapeutic strategy, genetic testing could provide additional information for a better understanding of the etiology and a more targeted treatment, especially in patients with persistent seizures and a rapidly progressive form of MS. Over the past decade, emerging evidence has indicated that genetic etiologies are frequently implicated not only in epilepsy but also in autoimmune disorders, with recent studies identifying specific genes that increase the risk of multiple sclerosis (MS). The earliest genetic associations identified with multiple sclerosis (MS) were within the human leukocyte antigen (HLA) gene family—notably the HLA-DRB1*15:01 allele. Subsequently, genome-wide association studies (GWAS) have uncovered more than 200 additional non-HLA single-gene variants linked to an increased susceptibility to MS. Lastly, RASopathies, particularly those involving the KRAS gene (Kirsten rat sarcoma viral oncogene homolog), seem to play an important role in the development of MS. Notably, this gene family has also been involved in epileptic disorders [42,43,44,45]. Future studies should integrate comprehensive genetic panels and autoimmune screening to identify potential predispositions or overlapping pathologies and new treatment options.

In summary, pediatric patients with MS and epilepsy form a distinct subset that carries significant clinical implications and that requires careful, individualized attention. Our study highlights the critical need for neurologists who treat pediatric multiple sclerosis patients to maintain acute awareness of their patients’ conditions. Monitorization for subtle seizure symptoms is mandatory, because immediate treatment greatly improves patients’ quality of life and neurological results.

The intersection of a demyelinating autoimmune disorder and a neurological excitability disorder (epilepsy) calls for an interdisciplinary approach. The optimal coordination of disease-modifying therapies and anti-seizure treatments also requires a team-based approach. Holistic treatment approaches must address both the cognitive and the psychosocial impacts of these two medical conditions. The treatment protocol for epilepsy in patients with MS requires careful consideration of both neurological deficits and concurrent pharmaceutical medications. Patients who exhibit increased sensitivity to side effects or who take multiple central nervous system drugs will require dosage adjustments for their anticonvulsive medications. The MS care team and the epilepsy team should work together closely to detect any negative side effects from drug interactions.

Such an interdisciplinary care model might involve setting up combined clinics or case conferences that include pediatric MS specialists alongside epileptologists and, as necessary, neuropsychologists and rehabilitation therapists.

A better understanding of the pathophysiological crosstalk between MS and epilepsy in children could inform more effective therapeutic strategies—such as the early aggressive treatment of MS to prevent inflammatory insults that might trigger seizures and the judicious selection of AEDs that do not negatively interact with the demyelinating process.

## 5. Conclusions

The group of pediatric MS patients who develop epilepsy represents a unique population that displays various etiologies and clinical trajectories that have not yet been fully understood. Our single-center experience highlights that these children warrant special attention and further investigation to better delineate the particularities of this overlap. The development of tailored treatment strategies for both conditions relies on an improved understanding of their interaction. When seizures arise alongside demyelinating diseases like MS, the investigation must include a thorough review of standard pediatric epilepsy causes—especially genetic ones. The simultaneous presence of these conditions may result from coincidental factors or multiple causative elements. We live in an era of expanding genetic and immunological insights, and applying these to cases of MS–epilepsy comorbidity will enhance our understanding of the disease mechanisms. In our cohort, epilepsy appeared to be associated with a higher risk of accumulating disability (rising EDSS scores) over time in some children with MS. This suggests that the occurrence of seizures in a pediatric MS patient may portend a more aggressive disease course and necessitates careful monitoring and potentially stronger treatment approaches.

## Figures and Tables

**Table 1:** Seizures and epilepsy characteristics and outcomes.

Patient No.	Age at sz Onset	Time in Relation to MS Onset	Time Between sz Onset- and MS Onset	Time Between MS Onset and sz Onset	Seizure Aspects	Seizure Duration	Seizure Frequency	Epilepsy Diagnosis	AED Treatment	Seizure Control	Personal and Family History
P1	9 y 7 mo	after	NA	9 mo	Left focal motor SE, +LOC, at the end	3–4 h	Only 1 SE, awake	NO, acute, probable autoimmune	LEV 6 mo	YES, ~7 y, no AED at present	Neg
P2	4 y 6 mo	before	5 y 8 mo	NA	Type 1: Focal se generalized (head + eyes right deviation, oral automatism, gen hypertonia+/−clonic mov Type 2: myoclonic–atonic Type 3: absence of sz; rare GTC or focal sec gen	Type 1 <1′ Type 2 1–2” Type 3 5–10” absence of sz; 1–5′ GTC	Type 1 3/day, daily, awake and asleep Type 2 5–10/day, daily, awake Type 3 3–10/day, 2–3 times/week, awake; GTC/focal 2–3/year	YES, non-syndromic, probable genetic-autoimmune	Type 1 VPA, then * ACTH Type 2 VPA+CNZ, * ACTH Type 3 +CBZ, +LEV, –CBZ	Type 1 Yes, after ACTH Type 2 Yes, after ACTH Type 3 NO, 2–4 sz/day VPA+CNZ+LEV at present	6 FS, between 2 and 4 y; mild cognitive decline; + some non-epileptic (reactive) events
P3	10 y 1 mo	before	6 y 5 mo	NA	Type 1: Generalized Type 2: Focal+/− sec gen(tinnitus/diplopia, headache, left mouth dev, L limb dystonic posture, +/−gen hypertonia and clonic mov, headache +/− vomiting after sz)	Type 1 1–2′ Type 2 1–2′	Type 1 1/5–6 mo, awake Type 21/1–2 mo, most of them awake	YES, non-syndromic, probable genetic–autoimmune	Type 1: VPA Type 2 + LEV + CBZ, + LTG, -CBZ	NO, sz freq stable in the last 3 y ** LEV+VPA+ LTG+CBZ at present	1 parent with epilepsy (adolescence); 2 FS before 2 y
P4	13 y 1 mo	before	1 y 8 mo	NA	GTCsz	1–2′	2 GTC sz, 2y apart, from awake	YES, GIEs (JME vs. GTCA)	LEV	YES, 1 y, LEV at present	Neg
P5	6 y 5 mo	before	4 y 9 mo	NA	Right focal motor sz (speech impairment, hypersalivation, R facio-brachial clonic mov, NO LOC)	<1′	2 focal sz, 2 mo apart, before sleep	YES, SeLECTS	VPA 2.5 y	YES,~12 y, no AED at present	1 parent with MS (adult onset)
P6	15 y 3 mo	after	NA	2 wks	Focal +/− sec gen sz 1st: nausea, dizziness, R head dev, RUL hypertonia + clonic mov, no LOC, 5 min aphasia 2nd: nausea, L head dev, gen hypertonia and clonic mov, left hemibody transient paresis	<1′, 1 asleep, 1 awake	2 sz, 4 days apart	YES, non-syndromic, probable auto-immune	LEV 2y	YES, ~3 y, no AED at present	Neg

P—patient; MS—multiple sclerosis; NA—not applicable; Neg—negative; AED—anti-epileptic drug; L—left; R—right; SE—status epileptic; LOC—loss of consciousness; RUL—right upper limb; FS—febrile seizure; focal sec gen—focal secondary generalized; sz—seizure; dev—deviation; mov—movement; LEV—levetiracetam; VPA—valproic acid; CBZ—carbamazepine; LTG—lamotrigine; CNZ—clonazepam; GTC—generalized tonic–clonic; GIEs—generalized idiopathic epilepsies; JME—juvenile myoclonic epilepsy; GTCA—generalized tonic–clonic alone; SeLECTS—self-limited epilepsy with centro-temporal lobe; ACTH *—unknown exact dosage and frequency, 1–2 mo duration, in another clinic; ** LEV + VPA + LTG + CBZ: LEV 2 g/day, VPA 500 mg/day (decreased from 1000 mg/day because of LTG; when LTG reaches 200 mg/day, try to decrease). Note: All AEDs were given in therapeutic doses according to the recommendations in the package leaflet and the international guidelines in force.

**Table 2:** Epilepsy in POMS as risk factor for higher last EDSS score 1.

Chi-Squared Test	Value	df	*p*
Χ^2^	7.536	1	0.006
Χ^2^ continuity correction	5.003	1	0.025
N	120		

**Table 3:** Epilepsy in POMS as risk factor for higher last EDSS score 2.

Log Odds Ratio				
		95% Confidence Interval		
	Log Odds Ratio		Upper	*p*
Odds ratio	2.124	0.364	3.884	
Fisher’s exact test	2.099	0.088	4.560	0.020

## Data Availability

All data supporting the findings of this study will be made available by the corresponding authors upon reasonable request.

## Abbreviations

The following abbreviations are used in this manuscript:

MS	Multiple Sclerosis
ILAE	International League Against Epilepsy
EDSS	Expanded Disability Status Scale
BBB	Blood–Brain Barrier
MRI	Magnetic Resonance Imaging
EEG	Electroencephalography
JASP	Jeffreys’s Amazing Statistics Program
AEDs	Anti-Epileptic Drugs
IGEs	Idiopathic Generalized Epilepsies
ICS	Isolated Clinical Syndrome
DMTs	Disease-Modifying Therapies
POMS	Pediatric-Onset Multiple Sclerosis
HLA	Human Leukocyte Antigen
GWAS	Genome-Wide Association Studies
KRAS	Kirsten Rat Sarcoma Viral Oncogene Homolog
ARR	Annualized Relapse Rate
HET	High-Efficacy Therapies

## Appendix B

**Table A1:** Detailed statistical analysis.

**Item**	**MS with Epilepsy Group** **(6 Patients)**	**MS Without Epilepsy Group (114 Patients)**	***p* Value (Where Appropriate)**
Gender (F:M)	3 F, 3 M	76 F, 38 M	
Age at MS onset	13.5 [5.25]	15 [3]	0.63
Age at MS diagnosis	14 [5]	15 [2]	0.55
Duration MS dg–DMT onset	1 [0.75]	1 [0]	0.057
Duration MS monitoring during study	3.5 [1]	3 [1]	0.034
ARR before treatment (1 y *)	1.5 [1] (6)	1 [1] (114)	0.68
ARR before treatment (2 y *)	1 [0.5] (2)	0.5 [0.5] (41)	0.77
ARR before treatment (3 y *)	0.83 [0.5] (2)	0.66 [0.66] (24)	0.79
ARR after treatment initiation (1 y *)	0 [0.75] (6)	0 [0] (114)	0.5
ARR after treatment initiation (2 y *)	0 [0.5] (5)	0.25 [0.5] (58)	0.82
ARR after treatment initiation (3 y *)	0.91 [0.41] (2)	0.33 [0.83] (30)	0.17
Actual/last EDSS score	1.25 [1.25]	0 [0]	0.003 ^#^

* indicates number of whole completed years under monitoring. # One-tailed Mann–Whitney U test indicating that the epilepsy group has a higher EDSS. Round brackets in ARR rows indicate the number of patients in each group that completed the whole period of monitoring indicated in the first column. ARR: annualized relapse rate.
